# Pharmacological and non-pharmacological therapeutic interventions for the treatment of spinal cord injury-induced pain

**DOI:** 10.3389/fpain.2022.991736

**Published:** 2022-08-24

**Authors:** Olivia C. Eller, Adam B. Willits, Erin E. Young, Kyle M. Baumbauer

**Affiliations:** ^1^Department of Cell Biology and Physiology, University of Kansas Medical Center, Kansas City, KS, United States; ^2^Department of Anesthesiology, University of Kansas Medical Center, Kansas City, KS, United States

**Keywords:** psychological intervention, diet intervention, exercise intervention, neuropathic pain, visceral pain, musculoskeletal pain

## Abstract

Spinal cord injury (SCI) is a complex neurophysiological disorder, which can result in many long-term complications including changes in mobility, bowel and bladder function, cardiovascular function, and metabolism. In addition, most individuals with SCI experience some form of chronic pain, with one-third of these individuals rating their pain as severe and unrelenting. SCI-induced chronic pain is considered to be “high impact” and broadly affects a number of outcome measures, including daily activity, physical and cognitive function, mood, sleep, and overall quality of life. The majority of SCI pain patients suffer from pain that emanates from regions located below the level of injury. This pain is often rated as the most severe and the underlying mechanisms involve injury-induced plasticity along the entire neuraxis and within the peripheral nervous system. Unfortunately, current therapies for SCI-induced chronic pain lack universal efficacy. Pharmacological treatments, such as opioids, anticonvulsants, and antidepressants, have been shown to have limited success in promoting pain relief. In addition, these treatments are accompanied by many adverse events and safety issues that compound existing functional deficits in the spinally injured, such as gastrointestinal motility and respiration. Non-pharmacological treatments are safer alternatives that can be specifically tailored to the individual and used in tandem with pharmacological therapies if needed. This review describes existing non-pharmacological therapies that have been used to treat SCI-induced pain in both preclinical models and clinical populations. These include physical (i.e., exercise, acupuncture, and hyper- or hypothermia treatments), psychological (i.e., meditation and cognitive behavioral therapy), and dietary interventions (i.e., ketogenic and anti-inflammatory diet). Findings on the effectiveness of these interventions in reducing SCI-induced pain and improving quality of life are discussed. Overall, although studies suggest non-pharmacological treatments could be beneficial in reducing SCI-induced chronic pain, further research is needed. Additionally, because chronic pain, including SCI pain, is complex and has both emotional and physiological components, treatment should be multidisciplinary in nature and ideally tailored specifically to the patient.

## Introduction

Between 249,000 and 363,000 Americans are currently living with spinal cord injury (SCI) (1), which represents a significant health care burden as this injury results in numerous long-term complications including, but not limited to, the loss of motor function, bowel and respiratory dysfunction, widespread inflammation, altered body composition and metabolic disorders (2). In addition, approximately 70% of individuals with SCI experience chronic pain (3), which significantly interferes with daily activities and negatively impacts quality of life. Current pharmacological interventions for pain management after SCI are largely ineffective, leading to individuals seeking alternative therapies. Studies have shown that 55–63% of individuals with SCI-induced chronic pain have tried non-pharmacological therapies (4, 5). These findings indicate that there is a need for translational research focused on the development of non-pharmacological therapies that reduce the incidence of SCI-induced pain and improve the quality of life of individuals with SCI.

One of the most common types of pain seen after SCI is neuropathic pain, which is caused by a lesion or disease of the somatosensory nervous system and can present at or below the level of injury. Neuropathic pain is frequently reported as the most severe type of pain in individuals with SCI (6), is often rated amongst the most significant patient complaints (7), and is resistant to current medical treatment (8). Neuropathic pain is usually described as sharp, shooting, or burning pain. The onset of at-level neuropathic pain generally occurs sooner (average 1.2 years) than below-level neuropathic pain (average 1.8 years) (6). The mechanisms underlying neuropathic pain are not fully defined, however changes in the periphery, spinal cord, and brain have all been implicated (9).

Conversely, the other most common type of pain after SCI is nociceptive pain, which is caused by actual or threatened damage to non-neuronal tissue and is due to activation of nociceptors. Nociceptive pain after SCI includes musculoskeletal and visceral pain. Musculoskeletal pain presents as tenderness to palpation of musculoskeletal structures, limitation of movement, and muscle spasticity. It has an average time of onset of 1.3 years and is usually described as a dull and aching pain, which originates from injury to bones, ligaments, muscles, and intervertebral disk (6). During the acute phase, musculoskeletal pain is often associated with structural damage that has not yet healed while the chronic phase is related to abnormal loading of joints associated with objects such as wheelchair or crutch use or sustained abnormal postures resulting from the SCI. Shoulder and back pain are often seen in individuals with SCI caused by unusual demands placed on the upper limbs and muscle weakness or tension from limited mobility and continued sitting.

The function of visceral organs can also be impaired below the level of injury (10). Patients with SCI above thoracic level 10 (T10) often develop neurogenic bowel and bladder as secondary complications that contribute to reduced quality of life and impaired rehabilitation (11–13). In total 80% of patients with SCI exhibit some degree of bladder dysfunction (14) and 60% develop bowel dysfunction (15). These patients report the loss of bowel and bladder function as their highest priority of concern over below-level pain and locomotor impairment. The symptoms of neurogenic bowel and bladder include but are not limited to motility/micturition defects, constipation, fecal and urinary incontinence, and visceral pain (15–23). Visceral pain is defined by the perceived origin in deeper structures of the abdomen, thorax, and pelvis and is often difficult to localize. This type of pain is primarily described as dull and/or cramping (24, 25). Visceral pain has the longest time to onset of all post-SCI pain categories, beginning an average of 4.2 years after SCI (6). Though rates of visceral pain after SCI are lower than musculoskeletal or neuropathic pain, this type of pain still occurs in up to 60% of patients during their lifespan and is most often described as severe or excruciating (6). In addition to unrelenting visceral pain, neurogenic bowel can be particularly disadvantageous to patient welfare given that complications of neurogenic bowel (i.e., autonomic dysreflexia, septicemia, etc.) account for the most prevalent causes of emergency medical treatment during the chronic stage of SCI care (26–29).

Finally, nociplastic pain is a third type of chronic pain that has been recently defined as pain that arises from altered nociception despite no clear evidence of actual or threatened tissue damage causing activation of peripheral nociceptors. To our knowledge there are no published studies describing nociplastic pain in SCI. However, as nociplastic pain often occurs with other forms of chronic pain (30) it may contribute to the diagnosis of neuropathic or nociceptive pain in individuals with SCI. Further work needs to be done to explore this topic.

## Types of pharmacological and procedural therapies for SCI pain

SCI-induced chronic pain has both physical and emotional aspects, making it complex to treat. Additionally, multiple types of pain can present in a single individual and each type of pain can be influenced by behavior and environmental factors. Therefore, a multidisciplinary approach that incorporates a variety of therapeutic interventions may be most effective by targeting each of these domains simultaneously. Individuals with SCI often state that pharmacological treatments do not work in treating their pain and they prefer to explore non-pharmacological interventions to avoid unwanted side effects (4, 5). There is a growing body of evidence in both pre-clinical and clinical literature on the use of non-pharmacological treatments for SCI pain (discussed in the next section), however, the need for continued work in this domain is gaining more traction. Currently, pharmacological and procedural therapies are the most commonly used for the different types of SCI-induced chronic pain. Pharmacological therapies generally fall into 5 classes of drugs: non-steroidal anti-inflammatory (NSAIDs), anticonvulsants, anti-depressants, muscle relaxants, and opioids. Each drug class works through a different mechanism in treating SCI pain and all have varying side effects. Procedural therapies include surgical intervention, and electrical stimulation.

### NSAIDs

NSAIDs such as Ibuprofen, Aspirin, and Naproxen are used to reduce inflammation, which is commonly increased after SCI at the site of spinal injury, at locations of secondary injuries, and systemically. Specifically, NSAIDs block cyclooxygenase, an enzyme involved in the process of converting arachidonic acid into prostaglandins (31). Prostaglandins are increased after tissue injury and contribute to inflammation through alterations in blood vessel dilation as well as increased vascular permeability which both work to induce edema (32). In addition, prostaglandins sensitize nociceptor terminals, which leads to increased neuronal firing that contributes to pain sensation (32). Side effects of long-term NSAID use include increased risk of dyspepsia, peptic ulcer disease, generalized upset stomach, bleeding problems, and renal dysfunction (33).

### Anticonvulsants and antidepressants

Anticonvulsants and antidepressants have been investigated for their efficacy in treating SCI pain due to their use in other neuropathic pain disorders (34). The most common anticonvulsants used to treat SCI pain include gabapentin, pregabalin, lamotrigine, and carbamazepine. Anticonvulsants are thought to improve pain after SCI through multiple mechanisms including suppression of abnormal neuronal excitability, increasing gamma-aminobutyric acid (GABA) inhibition, and modulating sodium and calcium channel activation (35). Side effects of anticonvulsants include dizziness, sleepiness, and nausea. Anti-depressants used after SCI include selective serotonin reuptake inhibitors (SSRIs), mixed serotonin-noradrenaline reuptake inhibitors (SNRIs), and tricyclic antidepressants (TCA). Generally, antidepressants work by inhibiting serotonin and/or norepinephrine reuptake which reduces neuronal hyperexcitability. TCAs have been shown to block sodium channel activation, induce opioid receptor activity, and antagonize the NMDA receptor for glutamate (35). Overall, the benefit of TCAs in treating pain lies in their ability to prevent cellular activity through inhibition of excitatory ion channels, thereby reducing nociceptor signaling. The most common antidepressants administered for SCI pain are the TCAs amitriptyline or imipramine, however when studied in a randomized controlled trial there were no differences compared to placebo (benztropine mesylate) in either pain intensity or pain-related disability (36). Although TCAs seem to be more effective than other pharmacological drugs in treating SCI pain, they come with more severe side effects which include sleepiness, dizziness, dry mouth, constipation, and nausea.

### Muscle relaxants

After SCI, decreased muscle movement and reduced descending inhibition of motor reflexes causes tightening of muscles and spasms, which are regularly associated with pain (37). Muscle relaxants, most commonly baclofen and tizanidine, are used to reduce the spasticity. Baclofen is an allosteric modulator of GABA receptors (38), which reduces spasticity through inhibition of alpha motor neurons in the spinal cord. Tizanidine also inhibits motor neurons by acting as an agonist of alpha 2-adrenergic receptors. Side effects of these medications include sleepiness and confusion.

### Opioids

Opioids are one of the most effective treatments for acute pain and have been coined the standard of care for severe pain after trauma. Opioids are an agonist of the three major classes of opioid receptors: κ-opioid receptor (KOR), μ-OR (MOR), and δ-OR (DOR) (39). These receptors are located throughout the central and peripheral nervous systems in areas involved in the transmission and modulation of pain and when activated traditionally result in analgesia (39). The MOR is the most common pharmacological target, such as with morphine or tramadol (40). Activation of the MOR reduces the presynaptic release of GABA (41) while tramadol blocks the reuptake of serotonin and norepinephrine and its metabolite is a MOR agonist (42). In addition, MOR activation modulates calcium and potassium channels, both of which are involved in pain processing (43, 44). Specifically, MOR activation results in the inhibition of N-type voltage gated calcium channels (45), which are located in the superficial laminae of the dorsal horn and control calcium entry into the synapse (46). Blocking these channels prevents the release of neurotransmitters including glutamate, substance P, and calcitonin gene related peptide (CGRP) (46). MOR activation also results in the opening of G-protein-coupled inward rectifying potassium channels (44). These channels are also located in the superficial laminae and opening them decreases neuronal excitability (47).

Despite their efficiency in acute pain reduction, opioids are associated with negative effects on the gastrointestinal tract including constipation, reduced motility, and nausea. Given that SCI patients develop gastrointestinal defects like constipation (48, 49), opioids will exacerbate gastrointestinal symptoms and prolong time to recovery. Long-term usage can result in significant side effects including sedation, respiratory depression, and bradycardia, as well as risk of tolerance, dependence, and abuse. Chronic opioid use has also been shown to result in opioid-induced hyperalgesia limiting the effectiveness in treating pain (50, 51). In addition, there is evidence in pre-clinical literature that use of opioids during the first week following SCI can impair locomotor recovery (8). The significant side effects, lack of long-term efficacy, and substance use disorders developed by opioid usage highlight the critical need for development of other pharmacological or non-pharmacological agents in pain management after SCI.

### Surgical and procedural interventions

There are a limited number of individuals with SCI that benefit from surgical intervention for pain due to the destructive nature of surgery on already injured nervous system tissue. Surgery is used for neurological decompression, to stabilize spinal segments, and to correct spinal deformations (52). Neurological decompression can be used to relieve pressure on the spinal cord or nerves caused by herniated disc, bone fragmentation, or even carpal tunnel syndrome resulting from chronic wheelchair use. Decompression involves the removal of damaged structures that are compressing neural tissue to create space. Spinal fusion is used to stabilize spinal segments using an implantable device (e.g., rods, screws, plates) and/or arthrodesis of the joint, which includes fusion of the adjacent bones. Dorsal root entry zone (DREZ) lesioning is also used for SCI pain (53), which involves a laminectomy followed by the administration of high voltage electrical activity and generation of microthermal lesions within the dorsal horn along the length of the exposed cord. However, DREZ is only used for patients after other more conservative interventions are not sufficient. While this procedure results in pain relief in certain instances, dorsal root avulsion has also been shown to result in neuropathic pain (54).

Electrical stimulation is also used for treatment of SCI pain. Transcutaneous electrical nerve stimulation (TENS), stimulation of specific brain regions, and epidural electrical stimulation of the spinal cord have all been used (55–57). These nerve stimulators can be minimally invasive by transcutaneous implantation of electrodes (58, 59) or a surgical approach which allows for direct implantation of spinal cord stimulators (60, 61). Even though there have been effective results in clinical trials, there are complications because of the invasive quality of spinal cord stimulation implantation. Some reported complications include, but are not limited to discomfort, nerve damage, cerebrospinal fluid leaking, and deafferentation (62).

### Management of SCI pain using pharmacological and procedural treatments

It is estimated that as little as 1/3 of individuals with SCI-induced pain receive even a 50% reduction in pain from current pharmacological therapies (9). Pharmacological treatments for SCI pain differ primarily based on pain type (neuropathic, musculoskeletal, visceral) and severity. For example, first line pharmacological treatments for neuropathic pain include anticonvulsants [gabapentin and pregabalin (63)] and, less frequently, tricyclic antidepressants, although there is less evidence for their efficacy. Acetaminophen is considered first-line treatment for acute musculoskeletal pain followed by NSAIDs and may be used alongside a muscle relaxant like baclofen, when pain is accompanied by muscle spasticity (64) or corticosteroid injections to reduce inflammation. For persistent pain of all origins, a weak opioid, such as tramadol, is considered second-line treatment (9) while stronger opioids, such as morphine, are considered third-line treatment. However, long-term opioid use can paradoxically lead to increased pain and other problematic side effects such as constipation and opioid dependence or misuse (9). Opioids are one of the few pharmacological options used to treat visceral pain, yet can play a direct role in initiating constipation and cramps (48, 49). The mechanisms that initiate and maintain visceral pain after SCI remain unclear, and, with few mechanisms to target, few effective options for treatment and prevention exist. Pharmacological treatments are used to improve bowel motility (e.g., laxatives and antispasmodics) but they can have secondary side effects and, unfortunately, have no direct effect on visceral pain. Even with the breadth of pharmacological approaches, patients' pain is not well controlled.

The most frequently used medical procedural interventions for SCI-induced pain include transcutaneous electrical nerve stimulation (TENS), spinal cord stimulation, surgical corrections/decompression, and DREZ to address neuropathic and musculoskeletal pain conditions (55, 65). Bladder function after SCI may be improved with prescribed spinal cord stimulation (alone or with activity-based training regimens) (61, 66) and sacral neuromodulation has been proposed to manage bladder pain syndrome/interstitial cystitis though most studies are not specific to SCI (67).

Catheterization is commonly used to treat micturition defects but does not address bladder pain directly and can lead to mechanical damage and introduce bacteria leading to inflammation and exacerbating pain (68).

## Non-pharmacological treatments for SCI pain

Pain after SCI is a significant problem due to its prevalence and the difficulty to manage it. While pharmacological interventions can improve *acute* neuropathic and musculoskeletal SCI pain, efficacy is limited in treating acute visceral pain. Further, when any form of SCI pain becomes *chronic* it is even more difficult to treat. The use of non-pharmacological interventions to replace or compliment conventional medication-based treatment may improve patient neuropathic and musculoskeletal pain outcomes. However, there remains a paucity in the literature regarding nonpharmacological interventions specific to SCI-induced visceral pain and gastrointestinal/bladder disorders, limiting effective treatment development.

### Exercise therapy for SCI pain

A growing body of research indicates that exercise can have a positive impact on chronic pain disorders, including migraine, fibromyalgia, pelvic pain disorders, and chronic low back pain (69, 70), although it is important that the exercise regimen is specifically tailored to the individual so as not to trigger or worsen their pain. Similarly, preclinical and clinical studies on exercise interventions for the treatment of SCI-induced chronic pain have found positive results. Additionally, exercise has been shown to reduce anxiety and depression (71), which is often found to be co-morbid in individuals with chronic pain, including pain associated with SCI (72–74).

Preclinical studies usually measure evoked mechanical or thermal pain-like behavior on the hindpaw or tail in spinally injured rodents. Mechanical nociception is most commonly measured using von Frey monofilaments (75) while thermal nociception is most commonly inferred by measuring tail or paw withdrawal latencies following presentation of a noxious thermal stimulus (76, 77). In studies using exercise as a therapeutic intervention for SCI-induced pain in rodents, a running wheel or treadmill are frequently used. However, the start day, duration, and intensity of the exercise protocol varies between studies (78). Despite these differences, many studies have shown that exercise improves pain-like behavior in rodent models of SCI. Specifically, SCI-induced mechanical hypersensitivity has been shown to be improved by exercise when it is started during either the acute (79–85) or chronic phase of SCI (81, 86, 87). Similarly, studies have found that SCI-induced thermal hypersensitivity is also improved by exercise started during the acute (81, 83–85, 88) or chronic phase (81, 86, 87). For example, Dugan and Sagen (81) administered a T6 SCI to rats and started treadmill training either 5 (acute phase) or 21 (chronic phase) days post-injury. Training took place 5 times per week for 12 weeks. They found that treadmill training started at either time point was able to improve mechanical allodynia (measured with the von Frey method) and heat hyperalgesia (measured with the Hargreaves method). Interestingly, a follow up experiment with the same exercise parameters found that upon cessation of exercise, the positive effects of exercise on pain-like behaviors were maintained for 2 weeks. The same group also demonstrated that treadmill training started 4 weeks after SCI and continued for 2 years resulted in continued improvements in mechanical, heat, and cold hypersensitivity compared to non-exercised rats (87).

Like humans with SCI, spinally injured animals have deficits in locomotor ability, which can pose challenges for exercise interventions. To circumvent this, running wheels can be modified to make a continuous walking surface to prevent further injury (80)and treadmills can be used with partial weight support of the animal (83, 85, 89). In addition, the majority of the studies mentioned above use a moderate SCI model, that allows for some recovery of locomotor function over time. Exercise protocols can be adjusted based on the animal's recovery of function.

Although the studies described above demonstrate a positive effect of exercise on SCI-induced pain in rodents, it should be noted that exercise is not always effective. A few studies have shown that exercise has no effect on SCI-induced mechanical hypersensitivity (79, 90) or thermal hypersensitivity (79, 80, 82, 90). The differences in these studies could be due to differences in experimental parameters including the level of SCI, exercise start day, or the intensity and duration of exercise. For example, one group that uses a unilateral cervical SCI model found that while starting exercise (automated running wheels) during the acute period of injury improves pain behavior (80), starting during the chronic period does not (90). Another example comes from a study that found exercise (assisted treadmill stepping) worsened mechanical hypersensitivity (89) despite using the same injury severity and starting assisted treadmill stepping at the same time post-injury as a study that found positive results of exercise on pain behavior (83). In this instance, the differences between the two studies were the intensity and duration of their exercise regimen. The study that found exercise worsened SCI-pain behavior used assisted treadmill stepping at a speed of 11 cm/s for 10 min/day and 5 days a week for 8 weeks. In contrast, the study that found benefits of exercise used assisted treadmill stepping at a speed of 2.5–3.5 cm/s for 20 min/day and 7 days a week for 2 weeks. These differences highlight the importance of tailoring exercise parameters to each individual when implementing an exercise intervention in clinical settings for SCI pain.

Clinical studies on exercise therapy for SCI-induced pain usually implement aerobic and/or resistance training. Arm ergometry is a commonly used form of aerobic exercise that has shown positive effects on SCI-induced chronic pain (91–93). This form of exercise, also referred to as an arm cycle or arm crank, enables an individual to pedal with their arms on an exercise machine. In addition to improving aerobic capacity, arm ergometry can also strengthen muscles in the chest, back, arms, and core. Norrbrink et al., studied the effect of arm ergometer use for 10 weeks in individuals with neuropathic, musculoskeletal, and/or visceral pain (91). They found that pain ratings in individuals with neuropathic pain decreased and all but 1 individual with musculoskeletal pain reported no pain at the end of the study. Only one individual in this study had visceral pain and it was unaffected by exercise, but the intensity of their neuropathic and musculoskeletal pain decreased. Similarly, Hicks et. al., implemented a 9-month arm ergometry exercise program in individuals with SCI and found that participants had significantly less pain, stress, and depression as well as a greater quality of life (93). However, the authors did not specify what type of pain the participants had. A follow up study on these participants 3 months later revealed that exercise adherence and quality of life had significantly decreased while pain and stress both increased (94). These findings indicate that continued exercise is important in maintaining the positive effects on both pain and quality of life of individuals with SCI. Another study paired resistance training with arm ergometry for 3 months and found that exercised spinally injured individuals reported significantly less pain (type not specified), depression, and stress compared to non-exercisers (92).

While the effects of exercise on neurogenic bowel and visceral pain are currently unclear, it is typically prescribed for SCI patients as a form of motor rehabilitation. Body-weight-supported treadmill training has been shown to increase below-level motor reflex strengthening, neuronal regeneration, and reduced muscle atrophy (95–98). It has been suggested that neurogenic bowel symptom improvement could in parallel advance motor learning. This group showed that locomotor training in SCI patients reduced defecation time, improved voiding efficiency and pressure, and increased bladder capacity; on the other hand, neither standing training nor general exercise influenced neurogenic bowel or neurogenic bladder symptoms (99, 100). This suggests that improvements in below level spinal reflexes may contribute to neurogenic bowel and bladder improvement more effectively than non-targeted exercise.

An interesting finding in several clinical studies is that the type of exercise, or general physical activity, is not necessarily a significant predictor of improvement in pain, stress, and anxiety after SCI. For example, an important study carried out by Lofgren and Norrbrink surveyed individuals with SCI-induced neuropathic pain to determine what therapies worked for decreasing their pain (101). Through diary entries and scientific interviews, they found that there was a significant disconnect in the treatment they were being offered for pain (usually drugs with unwanted side effects) and what actually worked to reduce their pain. One therapy described by multiple individuals that reduced their pain and improved their quality of life was physical activity. There was a range of physical activity among the participants (e.g., gardening, swimming, strength training, and high intensity exercise), indicating that it is important for physical activity as a therapy to be specifically tailored to an individual's capabilities and needs. A different study measured neuropathic pain scores before and after bouts of exercise in individuals with reported below-level neuropathic pain (102). This study was unique in that the pain questionnaires were completed in the individual's natural environment through a cell phone application, not in a laboratory setting. They found that participants reported a significant decrease in neuropathic pain following completion of at least one bout of exercise. Two participants did report higher pain scores after one of their bouts of exercise, and the authors speculate that intensity of the exercise may be a key factor in lowering pain scores and that more research needs to be done in this area to draw any definite conclusions. Finally, a third study determined that individuals with SCI that preformed the most sports activity (more than 3 times a week) had the lowest depression and anxiety scores and the highest score of vigor compared to less active individuals (103). Interestingly, the mode of sport did not significantly affect the results.

There are multiple mechanisms involved in mediating exercise-induced analgesia including activation of the endogenous opioid system, release of serotonin, and endocannabinoid signaling (104). Endogenous opioids are increased systemically following exercise (105) and opioidergic neurons are expressed in the rostral ventromedial medulla (RVM) (106), an important area of the brainstem involved in pain modulation. Additionally, exercise-induced analgesia can be at least partially reversed by administration of opioid receptor antagonist, naloxone, in both clinical (107, 108) and preclinical studies (109, 110). However, other studies have found that naloxone administration did not influence exercise-induced analgesia (111, 112), suggesting the involvement of other mechanisms. Serotonergic neurons are also located in the RVM (106) and serotonin levels are increased in the brain and brainstem in preclinical models after swimming or treadmill running (113–115). Further, systemic depletion of serotonin using para-chlorophenylalanine methyl ester prevents exercise induced analgesia (115). Clinical (116) and pre-clinical work (117) have also shown that endocannabinoids are increased following exercise and that endocannabinoid receptors in areas of the brain involved in pain-modulation, such as the periaqueductal gray, are activated following exercise (118). Additionally, the administration of cannabinoid receptor inverse agonists was shown to prevent both resistance and aerobic exercise induced antinociception (117, 118). Neurotrophic factors are also suggested to play a role in exercise induced-analgesia. For example, a pre-clinical study demonstrated that exercise (body weight-supported treadmill training) increased tropomyosin-related kinase B (TrkB) and glutamic acid decarboxylases (key enzymes in the synthesis of the inhibitory neurotransmitter GABA) in the spinal cord (85). They found that blocking TrkB signaling inhibited exercise-induced analgesia and decreased levels of glutamic acid decarboxylases. Another group found that the glial cell-line derived neurotrophic factor (GDNF) family of ligands could play a role in exercise-induced analgesia following SCI. Specifically, they found that SCI-induced neuropathic pain was associated with decreased levels of GDNF and artemin in the spinal cord and DRG and increased sprouting of GDNF responsive afferents in the spinal cord dorsal horn. Exercise following SCI reduced pain and maintained GDNF and artemin levels as well as prevented sprouting of GDNF responsive afferents (80). Although the aforementioned mechanisms are centrally mediated, exercise can also have effects in the periphery including the reduction of widespread inflammation. Exercise is known to reduce inflammation (119) through multiple mechanisms including increasing the release of interleukin-6 (IL-6) which subsequently increases the anti-inflammatory cytokine IL-10 and also the IL-1 receptor antagonist, down regulating pro-inflammatory cytokine expression, and reducing pro-inflammatory monocytes in circulation.

### Diet interventions for SCI pain

Individuals with SCI often experience chronic inflammation, which can contribute to the development of chronic pain (120). Specifically, SCI patients have increased circulating pro-inflammatory cytokines IL-1β, IL-6, IL-2, tumor necrosis factor (TNF)-α, and interferon gamma (IFN-γ) (121, 122). Increased widespread inflammation is also known to be present in spinally injured rodents (123, 124). These pro-inflammatory cytokines are known to cause hyperalgesia and contribute to the development of chronic pain. Caloric and nutrient requirements change significantly after SCI and are often not adequately met (125), which can contribute to chronic inflammation (126). There is limited research on dietary interventions for chronic pain after SCI, however, a few diets have started to be studied including the anti-inflammatory diet, the ketogenic diet, and the low fermentable oligosaccharides, disaccharides, monosaccharides and polyols (FODMAP) diet.

An anti-inflammatory diet, also commonly referred to as a Mediterranean diet, is high in fruits, vegetables, lean protein or plant based protein, whole grains, fiber, and healthy fats such as omega-3 fatty acids (127). Supplements with anti-inflammatory benefits are also common in an anti-inflammatory diet, including green tea, red wine, ginger, turmeric and antioxidants such as coenzyme Q10, tocopherols, and *n*- acetyl-cysteine. Certain foods are also eliminated from this diet, such as refined wheat and sugar products and hydrogenated oils. An anti-inflammatory diet has been shown to result in a widespread reduction in inflammation (128) and has reduced symptoms in other pain disorders in humans such as chronic headache, rheumatoid arthritis, inflammatory bowel disease, and osteoarthritis (129–131). Similarly, a pre-clinical study demonstrated that mice fed an anti-inflammatory diet displayed a shortened recovery time after an injection with the inflammatory solution complete Freund's adjuvant while another study showed that an anti-inflammatory diet improved chronic pain outcomes in a mouse model of early life stress (132). Finally, while there are no pre-clinical studies directly measuring the effect of an anti-inflammatory diet on neurogenic bowel/bladder and visceral pain, one study showed that the Mediterranean diet can rescue age-related motor learning in aged mice (133). Their findings suggest a mechanism by which neurogenic bowel outcomes could improve with a Mediterranean diet. Specifically, reduced large-molecule sugar intake and anti-inflammatory properties could lead to reduced gas/bloating as well as superoxide damage and systemic inflammation elimination. This could reduce visceral pain associated with SCI-induced neurogenic bowel.

The few clinical studies on the use of an anti-inflammatory diet for the treatment of SCI pain have demonstrated promising results. Allison et. al., measured neuropathic pain scores *via* the Neuropathic Pain Questionnaire and levels of circulating pro-inflammatory cytokines in participants with SCI after 12 weeks on an anti-inflammatory diet (134). They found that participants on the anti-inflammatory diet displayed significant reductions in neuropathic pain after 1 and 3 months on the diet compared to baseline assessment, while the participants in the control group displayed worse pain scores after 1 month and no change after 3 months. The treatment group also had significantly reduced pro-inflammatory cytokines at 3 months compared to baseline as well as compared to SCI participants on a control diet. An important follow up study in a subset of these participants examined the barriers and facilitators to adhering to this anti-inflammatory diet (135). They determined that the greatest barriers to adherence were social events, expense of the diet, lack of knowledge about the diet, and lack of motivation after the study was over. The greatest facilitators included family and peer support, health benefits from the diet (i.e., reductions in pain, edema, and improvements in cognition and mobility), autonomy over meal selection, and the implementation of adherence strategies (i.e., incorporating cheat days/meals to cope with the strict diet).

A ketogenic diet consists primarily of high fat (70–80%), moderate protein (10–20%), and very low carbohydrates (5–10%). Certain foods are eliminated from this diet including those with refined and whole grains and starchy vegetables and fruits, while foods that are high in saturated and unsaturated fat make up the majority of the diet. The ketogenic diet was first used in humans to treat epilepsy and has since been studied as a therapeutic intervention for different ailments including weight loss, dementia, amyotrophic lateral sclerosis (ALS), cancers, and metabolic disorders such as insulin resistance and type 2 diabetes (136). Importantly, pre-clinical literature provides several examples of consumption of a ketogenic diet resulting in improvements in allodynia and ongoing pain (137–140). Currently, clinical studies on the use of a ketogenic diet in individuals with SCI have focused more on improving functional recovery after SCI rather than pain outcomes. Yarar-Fisher et al., conducted a pilot study on the safety and feasibility of a ketogenic diet after SCI and they found a ketogenic diet for 5 +/- 2 weeks significantly improved upper extremity motor scores and lowered levels of the pro-inflammatory marker fibrinogen (141). They did not see any significant differences in sensory outcomes (light touch or pin prick). Similar results were observed in a rat model of cervical SCI, where 12 weeks on a ketogenic diet significantly improved forelimb motor function (142). More work needs to be done to explore the use of a ketogenic diet for the treatment of pain following SCI. However, based on findings from pre-clinical studies that used a ketogenic diet in other pain models (137–140), it could prove to be a promising treatment option. A potential pitfall for prescribing this diet is that it can be challenging to maintain due to the carbohydrate restriction.

A diet that could be specifically beneficial for the treatment of SCI-induced neurogenic bowel and visceral pain, is the low FODMAP diet, which eliminates fermentable oligosaccharides, disaccharides, monosaccharides, and polyols from the diet (143). These are short-chain sugars that are not easily broken down or absorbed by the colon and are thought to play a role in inducing gas and bloating/gastrointestinal distention. The low FODMAP diet reduces intake of these sugars and has been shown to improve gastrointestinal symptoms but the direct effect(s) on visceral pain remain incompletely understood. In addition, probiotic dietary supplementation could also be beneficial in reducing SCI-induced neurogenic bowel symptoms. For example, a probiotic drink reduced the incidence of antibiotic-associated diarrhea in individuals with SCI (144). While this study did not measure visceral pain, other studies have shown that probiotics reduce visceral pain symptom severity scores in individuals with irritable bowel syndrome as well as reduce bloating and altered bowel movements (145). Probiotics exert their beneficial effects on the bowel through their antimicrobial properties and by stabilizing bowel permeability (146).

The type of diet consumed can have a direct effect on an individual's inflammatory status (147), which can influence pain. One mechanism that both anti-inflammatory and ketogenic diets can have a positive impact on SCI pain is by reducing inflammation. As described before, an anti-inflammatory diet is high in fruits, vegetables, lean or plant based protein, whole grains, and healthy fats (127). Fruits and vegetables contain important vitamins, minerals, and antioxidants. Studies have shown that the higher the consumption of fruits and vegetables, the lower the level of inflammation and oxidative stress (148, 149). The consumption of red meat is low in this diet because it can increase inflammation (150). However, there is evidence that grass-fed vs. grain-fed beef products differ in their inflammatory properties such that consuming grain-fed beef can lead to higher inflammation levels compared to consuming grass-fed beef. Specifically, grass-fed beef contains higher omega-3 fatty acids, antioxidants such as glutathione and superoxide dismutase, and lower fat content compared to grain-fed beef (151). White meats and fish also have anti-inflammatory properties (147). In addition, some fish are high in omega-3 fatty acids, which have been shown to reduce pain (152). Other foods high in omega-3 fatty acids include flax seeds, walnuts, and soy beans, all of which also have anti-inflammatory properties (147). Whole grains are important in this diet because they have a low glycemic index. A diet including foods with a low glycemic index was shown to be more effective in reducing chronic inflammation (measured as C-reactive protein) compared to a diet including foods with high glycemic index (153). This study also demonstrated that high glycemic peaks was a contributor to increased oxidative stress. A ketogenic diet has also been shown to reduce inflammation in preclinical and clinical studies. For example, the consumption of a ketogenic diet increases circulating β-Hydroxybutyrate, which has been shown attenuate neuroinflammation in a rat model of SCI (137). Additionally, a ketogenic diet causes the body to utilize ketones instead of glucose for cellular energy, and ketone metabolism produces fewer reactive oxygen species and free radicals than glucose metabolism (154). Finally, ketone metabolism elevates adenosine (155), which is known to be anti-inflammatory (156).

### Heat and cold therapy for SCI pain

Manipulation of temperature by exposure to heat and cold has been theorized to help with recovery following SCI. Most studies that report the use of heat treatment [i.e., application of heat to the site of SCI pain, or whole-body heat treatment (hot tub or sauna)] for SCI pain are patient surveys. To our knowledge, no studies have been conducted to specifically examine the use of heat treatment for SCI pain. This is surprising given that heat is often reported as one of the top non-pharmacological treatments that individuals with SCI find is efficacious in reducing their pain (4, 157, 158). For example, Budh and Lundeberg used mailed questionnaires to determine the non-pharmacological treatments that were preferred by individuals with SCI-induced pain (4). Their results revealed that 90 of the 100 participants experienced neuropathic and/or nociceptive pain and that heat treatment was one of the most effective therapies for pain relief in this population. Specifically, 22 individuals had tried heat as a therapy for their pain and 77% reported that it was successful. In a similar patient survey by Ravenscroft et al., they found that 115 out of 146 individuals with SCI reported having pain (157). 80 of the individuals with pain reported having two or more different types of pain, but the pain types were not specified. They found that 21% of their sample population reported that heat treatment was one of the most effective interventions for pain relief. Finally, Tsai et al., identified effective non-pharmacological treatments in a population of individuals specifically with non-neuropathic SCI pain (158). They found that 190 of 391 participants reported 1 or more non-neuropathic pain area, most commonly located in the shoulders, back, knee, and hip. Heat therapy was one of the most frequently used non-pharmacological therapies (29%) and was said to be one of the most helpful treatments for overall non-neuropathic pain (80% of painful locations). Although the mechanisms underlying the effectiveness of heat in reducing SCI pain have not been studied, heat is known to improve blood flow and circulation, reduce inflammation, and decrease muscle and joint stiffness. Heat treatment reduces inflammation, in part, by increasing levels of heat shock proteins, which produce anti-inflammatory cytokines (159) and by improving circulation (160). In addition, decreasing muscle/joint stiffness is especially important in improving musculoskeletal pain, a common problem in individuals with SCI.

While heat therapy is often used after SCI-induced pain is established, cold therapy is usually administered in SCI patients during the very acute time period following SCI (161). Specifically, many studies have used therapeutic hypothermia within hours after injury using a cooling catheter placed in the femoral vein, a spinal cord cooling saddle, or applying cold saline to the spinal cord (161, 162). These studies reveal varying results but some show that hypothermia following SCI can result in functional improvement (162). Pre-clinical studies have found that hypothermia within hours of injury can improve locomotor function and decrease lesion severity (163). However, to our knowledge no clinical or pre-clinical studies have evaluated the development of pain following SCI in patients that have been exposed to therapeutic hypothermia, demonstrating a gap in knowledge.

### Acupuncture and vagal nerve stimulation for SCI pain

Acupuncture is another non-pharmacological therapy used for treating chronic pain disorders. It involves stimulation of certain parts of the body (acupoints) by insertion of thin needles through the skin. A recent meta-analysis concluded that acupuncture has beneficial effects in individuals with chronic pain disorders such as migraine, osteoarthritis, and musculoskeletal disorders and that these effects persist over time (164). However, there are arguments in the literature regarding the efficacy of acupuncture (165). Nayak et al., studied the use of acupuncture in individuals with SCI-induced pain and found that approximately half (*n* = 10) of the participants showed significant improvements in pain intensity after 7.5 weeks of treatment (166). The authors suggest that the location of pain could determine whether acupuncture is effective, as the subset of participants that benefited from acupuncture had chronic pain located above the level of SCI. Acupuncture has also been shown to improve symptoms of SCI-induced neurogenic bowel (167) and bladder (168). It is hypothesized that acupuncture treatment reduces SCI pain through multiple mechanisms including reduction of inflammation through release of antioxidant factors, inhibition of neuronal apoptosis, and increasing expression of neurotrophins (169).

Acupuncture can also stimulate the vagus nerve (170), which is important in conveying information from visceral and somatic regions, is the major component of the parasympathetic nervous system, and is important in regulating inflammation (171). Electrical stimulation can also be used on the vagus nerve, and this form of stimulation has been shown to influence production of inflammatory cytokines in both pre-clinical (172) and clinical settings (173). Electrical vagal nerve stimulation was also shown to attenuate visceral pain measured using colorectal distension (174). Interestingly, individuals with SCI have reduced vagal tone, suggesting vagal nerve stimulation could be beneficial in this population. Indeed, Chen et al., used electrical vagal nerve stimulation in spinally injured rats and found that it improved functional recovery, reduced tissue damage, and reduced the release of pro-inflammatory cytokines (TNFα, IL1 β, and IL6) and increased levels of anti-inflammatory cytokine IL10 (175), however pain was not assessed.

### Psychological interventions for SCI pain

Psychological factors, such as attitude, emotions, and coping strategies, can have a substantial impact on how an individual experiences pain. Chronic pain, including SCI-induced chronic pain, is often associated with mood disorders such as depression and anxiety (72–74). It is estimated that depression and anxiety are experienced in around 22% of the SCI population (74). Further, each of these co-morbid disorders (depression, anxiety, and chronic pain) amplify the others and significantly impact quality of life (73). Psychological therapies, such as cognitive behavioral therapy, mindfulness-based interventions, and meditation have been successfully used as therapeutic interventions in other chronic pain disorders (176–179). Though the exact physiological mechanisms of these therapies are unclear, the intended purpose is to relax the individual to reduce autonomic output (i.e., heart rate variability, respiratory rate, etc.), encourage pain distraction, and to address maladaptive emotional responses to pain (180, 181). Incorporating these types of therapies into a pain management plan and creating a multidisciplinary approach has been shown to help individuals manage their chronic pain (182). A significant benefit of psychological interventions is that there are few to zero unwanted side effects.

Cognitive behavioral therapy consists of educating patients about their pain, techniques on how to cope with their pain, such as relaxation training, and how to implement these cognitive coping techniques in real-life situations (183). This form of psychological therapy has been shown to successfully reduce depression and anxiety symptoms in individuals with SCI (184–187). Several studies have examined if cognitive behavioral therapy is a useful treatment for reducing pain after SCI (186–189). Heutink et al., carried out a multicenter randomized control trial on the use of cognitive behavioral therapy for 10 weeks in individuals with SCI-induced neuropathic pain (188). They assessed pain intensity and pain-related disability as well as anxiety, depression, and life satisfaction before treatment and 3- and 6-months after the intervention started. They found that the intervention group, but not the control group, displayed a significant decrease in pain intensity and pain-related disability at the 3-month timepoint. The intervention group also displayed a decrease in anxiety and an increase in participation in activities at the 3- and 6-month timepoints compared to baseline. Importantly, the participants stated they would recommend this type of treatment program to others but suggested it be offered earlier following SCI. Burns et al., measured the influence of 10-weeks of cognitive behavioral therapy paired with either group exercise or guided relaxation in individuals with SCI-induced neuropathic pain. They found that although this treatment program did not reduce pain severity, it did help individuals cope with their pain, lessened pain interference with daily activities, and improved their sense of control (189). Many of the studies using cognitive behavioral therapy as a treatment for SCI-induced pain, anxiety, and/or depression suggest that “refresher” courses to reinforce skills learned during the treatment program are important to maintain efficacy of this treatment.

Mindfulness-based interventions are different from cognitive behavioral therapy as these interventions aim to facilitate present-moment awareness and acceptance, rather than attempting to change behavioral and psychological responses (190). A systemic review by Streijger et al., on the use of mindfulness-based interventions in individuals with SCI pain found a variation in results on this therapy's efficacy in the 5 studies reviewed (190). One study reported a significant reduction in SCI pain, while the others reported no change. Additionally, 4/5 studies reported a significant reduction in symptoms of depression while 3/5 reported reductions in anxiety. The authors suggest that while mindfulness might not improve SCI pain, it could be used to reduce incidence of depression and anxiety in individuals with SCI-induced co-morbid pain and mood disorders. However, two recent studies using different forms of mindfulness interventions [meditation (191) and yoga (192)] in individuals with SCI, demonstrated positive outcomes on pain, anxiety, and depression. Zanca et al., used a 4-week clinical meditation and imagery program, which included mindfulness, meditation, and guided imagery in individuals with chronic nociceptive and/or neuropathic SCI pain (191). They measured pain outcomes as well as depressive symptomology and perceived stress. Their results indicated that the intervention group showed a greater decrease in depressive symptomology and worst pain intensity over the last week and a greater increase in perceived control over pain. Although their results did not reach statistical significance, the authors note that it was a pilot study and that their sample size was small. Chalageri et al., used 1 month of *raja yoga*, which is a meditation technique, and found a significant decrease in numeric pain rating, anxiety and depression, and perceived stress scale in individuals with SCI that received the *raja yoga* treatment compared to those that received conventional rehabilitation (192). Additionally, they saw a significant increase in quality-of-life scores in the intervention group.

Finally, SCI patients that have developed neurogenic bowel and/or neurogenic bladder report psychological distress, anxiety, and embarrassment about their abdominal pain, constipation, incontinence, or need for catheterization. Prescribing cognitive behavioral therapy, meditation, or yoga/exercise may improve the mental strain of neurogenic bowel and neurogenic bladder inhibiting the hyperactive autonomic nervous system as suggested by functional gastrointestinal disorders models, such as irritable bowel syndrome.

## Conclusions

**Figure 1:**
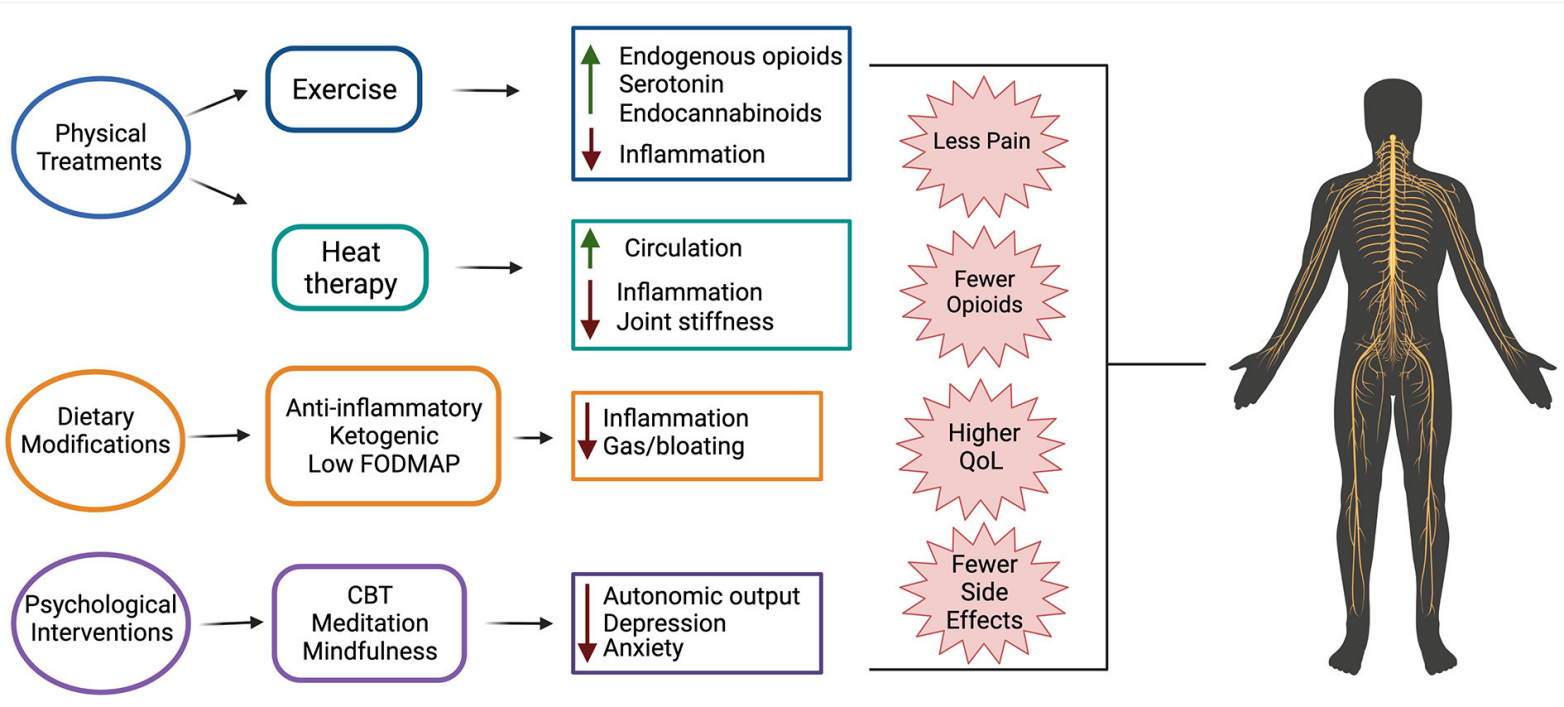
Non-pharmacological therapeutics for treating SCI pain and their suggested mechanisms of action. Evidence suggests that physical treatments (exercise and heat therapy), dietary modifications (anti-inflammatory, ketogenic, and low FODMAP diets), and psychological interventions (cognitive behavioral therapy (CBT), meditation, and mindfulness) are beneficial for use in individuals with SCI-induced pain. Use of these treatments results in fewer side effects than traditional pharmacological treatments, less opioid use, reduced pain, and overall higher quality of life.

Chronic pain is a significant problem for individuals with SCI, in part, because current pharmacological therapies are largely ineffective. This indicates a critical need for establishment of more efficacious therapies that can be used in lieu of or in combination with current drugs. Non-pharmacological therapies are often used by individuals with SCI pain that do not find relief from traditional pharmacological therapies. These therapies are appealing because they exert whole body effects, thereby targeting multiple mechanisms that contribute to SCI-induced pain (Figure 1). Non-pharmacological therapies are also generally safe to use and do not have negative off target effects that many pharmacological therapies do.

While many of these non-pharmacological therapies show promise in treating SCI-induced musculoskeletal or neuropathic pain, this review highlights a primary knowledge gap in the research on neurogenic bowel and bladder after SCI. These patients develop severe and unrelenting chronic abdominal pain along with bowel and bladder dysfunction. There is a critically important need to develop effective treatment options for these patients, though the complexity of these symptoms remains a barrier to effective treatment development. The few available non-pharmacological interventions suggested by this review and previous literature show promise yet are widely understudied. Each intervention appears to minimally rescue some but not all SCI-induced neurogenic bowel and neurogenic bladder symptoms, suggesting the need for a patient-specific multidisciplinary technique to cover all symptoms.

In addition, it should be noted that the majority of pre-clinical studies on SCI-induced pain have been carried out in female rodents. This is largely due to the fact that bladder expression after SCI is easier in female rodents, which results in fewer post-surgical complications (193). However, sex differences exist in many physiological processes, including pain and inflammation (194). This caveat should be taken into consideration when designing both pre-clinical and clinical studies examining the effects of therapies on SCI-induced pain.

In conclusion, although this review describes evidence of the beneficial effects of non-pharmacological therapies, these therapies are not always successful in all individuals with SCI pain. This highlights a critical need for development of precision pain treatment that matches a patient's pain phenotype (e.g., type of pain, severity, location) with a specific treatment strategy, which could include both pharmacologic and non-pharmacologic therapies to achieve the greatest benefit.

## Author contributions

OE and AW contributed to literature review, drafting, and revision of the manuscript. EY and KB contributed to integration of reviewed literature, drafting, and revision of the manuscript. All authors contributed to the article and approved the submitted version.

## Funding

This work was supported by a SCIRTS grant from the Craig H Nielsen Foundation (KB) as well as the Rita Allen Foundation (KB), R21NS104789-01A1 (KB) and the Kansas INBRE, P20 GM103418 (OE, KB, and EY).

## Conflict of interest

The authors declare that the research was conducted in the absence of any commercial or financial relationships that could be construed as a potential conflict of interest.

## Publisher's note

All claims expressed in this article are solely those of the authors and do not necessarily represent those of their affiliated organizations, or those of the publisher, the editors and the reviewers. Any product that may be evaluated in this article, or claim that may be made by its manufacturer, is not guaranteed or endorsed by the publisher.

## References

[1] Lasfargues JE Custis D Morrone F Carswell J Nguyen T. A model for estimating spinal cord injury prevalence in the United States. Paraplegia. (1995) 33:62–8. 10.1038/sc.1995.16 7753569

[2] Jensen MP Kuehn CM Amtmann D Cardenas DD. Symptom burden in persons with spinal cord injury. Arch Phys Med Rehabil. (2007) 88:638–45. 10.1016/j.apmr.2007.02.002 17466734 PMC2864010

[3] Cardenas DD Felix ER. Pain after spinal cord injury: a review of classification, treatment approaches, and treatment assessment. PMR. (2009) 1:1077–90. 10.1016/j.pmrj.2009.07.002 19797006

[4] Norrbrink Budh C Lundeberg T. Non-pharmacological pain-relieving therapies in individuals with spinal cord injury: a patient perspective. Complement Ther Med. (2004) 12:189–97. 10.1016/j.ctim.2004.10.003 15649832

[5] Nayak S Matheis RJ Agostinelli S Shifleft SC. The use of complementary and alternative therapies for chronic pain following spinal cord injury: a pilot survey. J Spinal Cord Med. (2001) 24:54–62. 10.1080/10790268.2001.11753556 11587436

[6] Siddall PJ McClelland JM Rutkowski SB Cousins MJ. A longitudinal study of the prevalence and characteristics of pain in the first 5 years following spinal cord injury. Pain. (2003) 103:249–57. 10.1016/S0304-3959(02)00452-9 12791431

[7] Kennedy P Lude P Taylor N. Quality of life, social participation, appraisals and coping post spinal cord injury: a review of four community samples. Spinal Cord. (2006) 44:95–105. 10.1038/sj.sc.3101787 16130026

[8] Woller SA Hook MA. Opioid administration following spinal cord injury: implications for pain and locomotor recovery. Exp Neurol. (2013) 247:328–41. 10.1016/j.expneurol.2013.03.008 23501709 PMC3742731

[9] Siddall PJ Middleton JW. Spinal cord injury-induced pain: mechanisms and treatments. Pain Manag. (2015) 5:493–507. 10.2217/pmt.15.47 26402151

[10] Widerstrom-Noga E. Neuropathic pain and spinal cord injury: phenotypes and pharmacological management. Drugs. (2017) 77:967–84. 10.1007/s40265-017-0747-8 28451808

[11] Coggrave M Norton C Wilson-Barnett J. Management of neurogenic bowel dysfunction in the community after spinal cord injury: a postal survey in the United Kingdom. Spinal Cord. (2009) 47:323–30. 10.1038/sc.2008.137 19015665

[12] Coggrave M Norton C Management of faecal incontinence and constipation in adults with central neurological diseases. Cochrane Database Syst Rev. (2013) CD002115. 10.1002/14651858.CD002115.pub4 24347087

[13] Lynch AC Antony A Dobbs BR Frizelle FA. Bowel dysfunction following spinal cord injury. Spinal Cord. (2001) 39:193–203. 10.1038/sj.sc.3101119 11420734

[14] Taweel WA Seyam R. Neurogenic bladder in spinal cord injury patients. Res Rep Urol. (2015) 7:85–99. 10.2147/RRU.S29644 26090342 PMC4467746

[15] Finnerup NB Faaborg P Krogh K Jensen TS. Abdominal pain in long-term spinal cord injury. Spinal Cord. (2008) 46:198–203. 10.1038/sj.sc.3102097 17621311

[16] Burke D Fullen BM Lennon O. Pain profiles in a community dwelling population following spinal cord injury: a national survey. J Spinal Cord Med. (2019) 42:201–11. 10.1080/10790268.2017.1351051 28738744 PMC6419620

[17] Burke D Fullen BM Stokes D Lennon O. Neuropathic pain prevalence following spinal cord injury: A systematic review and meta-analysis. Eur J Pain. (2017) 21:29–44. 10.1002/ejp.905 27341614

[18] Hulsebosch CE Hains BC Crown ED Carlton SM. Mechanisms of chronic central neuropathic pain after spinal cord injury. Brain Res Rev. (2009) 60:202–13. 10.1016/j.brainresrev.2008.12.010 19154757 PMC2796975

[19] Cruz-Almeida Y Martinez-Arizala A Widerstrom-Noga EG. Chronicity of pain associated with spinal cord injury: A longitudinal analysis. J Rehabil Res Dev. (2005) 42:585–94. 10.1682/JRRD.2005.02.0045 16586184

[20] Rekand T Hagen EM Gronning M. Chronic pain following spinal cord injury. Tidsskr Nor Laegeforen. (2012) 132:974–9. 10.4045/tidsskr.11.0794 22562333

[21] Levi R Hultling C Nash MS Seiger A. The Stockholm spinal cord injury study: 1. Medical problems in a regional SCI population. Paraplegia. (1995) 33:308–15. 10.1038/sc.1995.70 7644255

[22] Anderson KD. Targeting recovery: priorities of the spinal cord-injured population. J Neurotrauma. (2004) 21:1371–83. 10.1089/neu.2004.21.1371 15672628

[23] Nielsen SD Faaborg PM Christensen P Krogh K Finnerup NB. Chronic abdominal pain in long-term spinal cord injury: a follow-up study. Spinal Cord. (2017) 55:290–3. 10.1038/sc.2016.124 27502843

[24] Demirel G Yllmaz H Gencosmanoglu B Kesiktas N. Pain following spinal cord injury. Spinal Cord. (1998) 36:25–8. 10.1038/sj.sc.3100523 9471134

[25] Siddall PJ Taylor DA Cousins MJ. Classification of pain following spinal cord injury. Spinal Cord. (1997) 35:69–75. 10.1038/sj.sc.3100365 9044512

[26] Karlsson AK. Autonomic dysfunction in spinal cord injury: clinical presentation of symptoms and signs. Prog Brain Res. (2006) 152:1–8. 10.1016/S0079-6123(05)52034-X 16198689

[27] Lindan R Joiner E Freehafer AA Hazel C. Incidence and clinical features of autonomic dysreflexia in patients with spinal cord injury. Paraplegia. (1980) 18:285–92. 10.1038/sc.1980.51 7443280

[28] Jacob JE Pniak A Weaver LC Brown A. Autonomic dysreflexia in a mouse model of spinal cord injury. Neuroscience. (2001) 108:687–93. 10.1016/S0306-4522(01)00436-5 11738503

[29] Weiterer S Frick S Lichtenstern C Hug A Uhle F Weigand MA. Sepsis in mechanically ventilated patients with spinal cord injury: a retrospective analysis. Spinal Cord. (2019) 57:293–300. 10.1038/s41393-018-0217-5 30413803 PMC6477786

[30] Fitzcharles MA Cohen SP Clauw DJ Littlejohn G Usui C Hauser W. Nociplastic pain: towards an understanding of prevalent pain conditions. Lancet. (2021) 397:2098–110. 10.1016/S0140-6736(21)00392-5 34062144

[31] Zarghi A Arfaei S. Selective COX-2 inhibitors: a review of their structure-activity relationships. Iran J Pharm Res. (2011) 10:655–83. 24250402 PMC3813081

[32] Ricciotti E FitzGerald GA. Prostaglandins and inflammation. Arterioscler Thromb Vasc Biol. (2011) 31:986–1000. 10.1161/ATVBAHA.110.207449 21508345 PMC3081099

[33] Marcum ZA Hanlon JT. Recognizing the risks of chronic nonsteroidal anti-inflammatory drug use in older adults. Ann Longterm Care. (2010) 18:24–27. 21857795 PMC3158445

[34] Sindrup SH Jensen TS. Efficacy of pharmacological treatments of neuropathic pain: an update and effect related to mechanism of drug action. Pain. (1999) 83:389–400. 10.1016/S0304-3959(99)00154-2 10568846

[35] Finnerup NB Jensen TS. Spinal cord injury pain–mechanisms and treatment. Eur J Neurol. (2004) 11:73–82. 10.1046/j.1351-5101.2003.00725.x 14748766

[36] Cardenas DD Warms CA Turner JA Marshall H Brooke MM Loeser JD. Efficacy of amitriptyline for relief of pain in spinal cord injury: results of a randomized controlled trial. Pain. (2002) 96:365–73. 10.1016/S0304-3959(01)00483-3 11973011

[37] Taricco M Adone R Pagliacci C Telaro E. Pharmacological interventions for spasticity following spinal cord injury. Cochrane Database Syst Rev. (2000) 2000:CD001131. 10.1002/14651858.CD001131 10796750 PMC8406943

[38] Pin JP Prezeau L. Allosteric modulators of GABA(B) receptors: mechanism of action and therapeutic perspective. Curr Neuropharmacol. (2007) 5:195–201. 10.2174/157015907781695919 19305802 PMC2656813

[39] Rosenblum A Marsch LA Joseph H Portenoy RK. Opioids and the treatment of chronic pain: controversies, current status, and future directions. Exp Clin Psychopharmacol. (2008) 16:405–16. 10.1037/a0013628 18837637 PMC2711509

[40] Quirion B Bergeron F Blais V Gendron L. The delta-opioid receptor; a target for the treatment of pain. Front Mol Neurosci. (2020) 13:52. 10.3389/fnmol.2020.00052 32431594 PMC7214757

[41] Vaughan CW Ingram SL Connor MA Christie MJ. How opioids inhibit GABA-mediated neurotransmission. Nature. (1997) 390:611–4. 10.1038/37610 9403690

[42] Raffa RB Friderichs E Reimann W Shank RP Codd EE Vaught JL. Opioid and nonopioid components independently contribute to the mechanism of action of tramadol, an 'atypical' opioid analgesic. J Pharmacol Exp Ther. (1992) 260:275–85. 1309873

[43] Park J Luo ZD. Calcium channel functions in pain processing. Channels. (2010) 4:510–7. 10.4161/chan.4.6.12869 21150297 PMC3052250

[44] Ocana M Cendan CM Cobos EJ Entrena JM Baeyens JM. Potassium channels and pain: present realities and future opportunities. Eur J Pharmacol. (2004) 500:203–19. 10.1016/j.ejphar.2004.07.026 15464034

[45] Seward E Hammond C Henderson G. Mu-opioid-receptor-mediated inhibition of the N-type calcium-channel current. Proc Biol Sci. (1991) 244:129–35. 10.1098/rspb.1991.0061 1679547

[46] Zamponi GW Lewis RJ Todorovic SM Arneric SP Snutch TP. Role of voltage-gated calcium channels in ascending pain pathways. Brain Res Rev. (2009) 60:84–9. 10.1016/j.brainresrev.2008.12.021 19162069 PMC2692704

[47] Tsantoulas C McMahon SB. Opening paths to novel analgesics: the role of potassium channels in chronic pain. Trends Neurosci. (2014) 37:146–58. 10.1016/j.tins.2013.12.002 24461875 PMC3945816

[48] Grunkemeier DM Cassara JE Dalton CB Drossman DA. The narcotic bowel syndrome: clinical features, pathophysiology, and management. Clin Gastroenterol Hepatol. (2007) 5:1126–39. 10.1016/j.cgh.2007.06.013 17916540 PMC2074872

[49] Boland JW Boland EG. Pharacological therapies for opioid induced constipation in adults with cancer. BMJ. (2017) 358:j3313. 10.1136/bmj.j3313 28768637

[50] Lee M Silverman SM Hansen H Patel VB Manchikanti L. A comprehensive review of opioid-induced hyperalgesia. Pain Physician. (2011) 14:145–61. 10.36076/ppj.2011/14/145 21412369

[51] Mao J. Opioid-induced abnormal pain sensitivity: implications in clinical opioid therapy. Pain. (2002) 100:213–7. 10.1016/S0304-3959(02)00422-0 12467992

[52] Ragnarsson KT. Management of pain in persons with spinal cord injury. J Spinal Cord Med. (1997) 20:186–99. 10.1080/10790268.1997.11719468 9144608

[53] Nashold BS Ostdahl RH. Dorsal root entry zone lesions for pain relief. J Neurosurg. (1979) 51:59–69. 10.3171/jns.1979.51.1.0059 448420

[54] Aldskogius H Kozlova EN. Dorsal root injury-a model for exploring pathophysiology and therapeutic strategies in spinal cord injury. Cells. (2021) 10:2185. 10.3390/cells10092185 34571835 PMC8470715

[55] Norrbrink C. Transcutaneous electrical nerve stimulation for treatment of spinal cord injury neuropathic pain. J Rehabil Res Dev. (2009) 46:85–93. 19533522

[56] Bi X Lv H Chen BL Li X Wang XQ. Effects of transcutaneous electrical nerve stimulation on pain in patients with spinal cord injury: a randomized controlled trial. J Phys Ther Sci. (2015) 27:23–5. 10.1589/jpts.27.23 25642029 PMC4305569

[57] Cruccu G Garcia-Larrea L Hansson P Keindl M Lefaucheur JP Paulus W. EAN guidelines on central neurostimulation therapy in chronic pain conditions. Eur J Neurol. (2016) 23:1489–99. 10.1111/ene.13103 27511815

[58] Parittotokkaporn S Varghese C O'Grady G Svirskis D Subramanian S O'Carroll SJ. Non-invasive neuromodulation for bowel, bladder and sexual restoration following spinal cord injury: a systematic review. Clin Neurol Neurosurg. (2020) 194:105822. 10.1016/j.clineuro.2020.105822 32334284

[59] Niu T Bennett CJ Keller TL Leiter JC Lu DC. A proof-of-concept study of transcutaneous magnetic spinal cord stimulation for neurogenic bladder. Sci Rep. (2018) 8:12549. 10.1038/s41598-018-30232-z 30135433 PMC6105631

[60] Steadman CJ Grill WM. Spinal cord stimulation for the restoration of bladder function after spinal cord injury. Healthc Technol Lett. (2020) 7:87–92. 10.1049/htl.2020.0026 32754343 PMC7353924

[61] Redshaw JD Lenherr SM Elliott SP Stoffel JT Rosenbluth JP Presson AP. Protocol for a randomized clinical trial investigating early sacral nerve stimulation as an adjunct to standard neurogenic bladder management following acute spinal cord injury. BMC Urol. (2018) 18:72. 10.1186/s12894-018-0383-y 30157824 PMC6116487

[62] Guiho T Azevedo-Coste C Bauchet L Delleci C Vignes JR Guiraud D. Sacral anterior root stimulation and visceral function outcomes in spinal cord injury-a systematic review of the literature over four decades. World Neurosurg. (2022) 157:218–232. 10.1016/j.wneu.2021.09.041 34547528

[63] Attal N Mazaltarine G Perrouin-Verbe B Albert T. Medicine, and Rehabilitation, Chronic neuropathic pain management in spinal cord injury patients. What is the efficacy of pharmacological treatments with a general mode of administration? (oral, transdermal, intravenous). Ann Phys Rehabil Med. (2009) 52:124–41. 10.1016/j.rehab.2008.12.011 19909703

[64] Loubser PG Akman NM. Effects of intrathecal baclofen on chronic spinal cord injury pain. J Pain Symptom Manage. (1996) 12:241–7. 10.1016/0885-3924(96)00152-2 8898508

[65] Sindou M Mertens P Wael M. Microsurgical DREZotomy for pain due to spinal cord and/or cauda equina injuries: long-term results in a series of 44 patients. Pain. (2001) 92:159–71. 10.1016/S0304-3959(00)00487-5 11323137

[66] Gad PN Kreydin E Zhong H Latack K Edgerton VR. Non-invasive neuromodulation of spinal cord restores lower urinary tract function after paralysis. Front Neurosci. (2018) 12:432. 10.3389/fnins.2018.00432 30008661 PMC6034097

[67] Wang J Chen Y Chen J Zhang G Wu P. Sacral neuromodulation for refractory bladder pain syndrome/interstitial cystitis: a global systematic review and meta-analysis. Sci Rep. (2017) 7:11031. 10.1038/s41598-017-11062-x 28887515 PMC5591255

[68] Kinnear N Barnett D O'Callaghan M Horsell K Gani J Hennessey D. The impact of catheter-based bladder drainage method on urinary tract infection risk in spinal cord injury and neurogenic bladder: a systematic review. Neurourol Urodyn. (2020) 39:854–62. 10.1002/nau.24253 31845396

[69] Eller-Smith OC Nicol AL Christianson JA. Potential mechanisms underlying centralized pain and emerging therapeutic interventions. Front Cell Neurosci. (2018) 12:35. 10.3389/fncel.2018.00035 29487504 PMC5816755

[70] Daenen L Varkey E Kellmann M Nijs J. Exercise, not to exercise, or how to exercise in patients with chronic pain? Applying science to practice. Clin J Pain. (2015) 31:108–14. 10.1097/AJP.0000000000000099 24662498

[71] Byrne A Bryne DG. The effect of exercise on depression, anxiety and other mood states: a review. J Psychosom Res. (1993) 37:565–74. 10.1016/0022-3999(93)90050-P 8410742

[72] Bair MJ Robinson RL Katon W Kroenke K. Depression and pain comorbidity: a literature review. Arch Intern Med. (2003) 163:2433–45. 10.1001/archinte.163.20.2433 14609780

[73] Ullrich PM Lincoln RK Tackett MJ Miskevics S Smith BM Weaver FM. Pain, depression, and health care utilization over time after spinal cord injury. Rehabil Psychol. (2013) 58:158–65. 10.1037/a0032047 23713727

[74] Williams R Murray A. Prevalence of depression after spinal cord injury: a meta-analysis. Arch Phys Med Rehabil. (2015) 96:133–40. 10.1016/j.apmr.2014.08.016 25220943

[75] Chaplan SR Bach FW Pogrel JW Chung JM Yaksh TL. Quantitative assessment of tactile allodynia in the rat paw. J Neurosci Methods. (1994) 53:55–63. 10.1016/0165-0270(94)90144-9 7990513

[76] Hargreaves K Dubner R Brown F Flores C Joris J. A new and sensitive method for measuring thermal nociception in cutaneous hyperalgesia. Pain. (1988) 32:77–88. 10.1016/0304-3959(88)90026-7 3340425

[77] D'Armour WL Smith DL. A method for determining loss of pain sensation. J Pharmacol Exp Therap. (1941) 72:74–79.

[78] Palandi J Bobinski F de Oliveira GM Ilha J. Neuropathic pain after spinal cord injury and physical exercise in animal models: a systematic review and meta-analysis. Neurosci Biobehav Rev. (2020) 108:781–795. 10.1016/j.neubiorev.2019.12.016 31837360

[79] Brown AK Woller SA Moreno G Grau JW Hook MA. Exercise therapy and recovery after SCI: evidence that shows early intervention improves recovery of function. Spinal Cord. (2011) 49:623–8. 10.1038/sc.2010.167 21242998 PMC3230555

[80] Detloff MR Smith EJ Quiros Molina D Ganzer PD Houle JD. Acute exercise prevents the development of neuropathic pain and the sprouting of non-peptidergic (GDNF- and artemin-responsive) c-fibers after spinal cord injury. Exp Neurol. (2014) 255:38–48. 10.1016/j.expneurol.2014.02.013 24560714 PMC4036591

[81] Dugan EA Sagen J. An intensive locomotor training paradigm improves neuropathic pain following spinal cord compression injury in rats. J Neurotrauma. (2015) 32:622–32. 10.1089/neu.2014.3692 25539034

[82] Nees TA Tappe-Theodor A Sliwinski C Motsch M Rupp R Kuner R. Early-onset treadmill training reduces mechanical allodynia and modulates calcitonin gene-related peptide fiber density in lamina III/IV in a mouse model of spinal cord contusion injury. Pain. (2016) 157:687–97. 10.1097/j.pain.0000000000000422 26588690

[83] Tashiro S Shinozaki M Mukaino M Renault-Mihara F Toyama Y Liu M. BDNF induced by treadmill training contributes to the suppression of spasticity and allodynia after spinal cord injury via upregulation of KCC2. Neurorehabil Neural Repair. (2015) 29:677–89. 10.1177/1545968314562110 25527489

[84] Cheng X Yu Z Hu W Chen J Chen W Wang L. Voluntary exercise ameliorates neuropathic pain by suppressing calcitonin gene-related peptide and ionized calcium-binding adapter molecule 1 overexpression in the lumbar dorsal horns in response to injury to the cervical spinal cord. Exp Neurol. (2022) 354:114105. 10.1016/j.expneurol.2022.114105 35525308

[85] Li X Wang Q Ding J Wang S Dong C Wu Q. Exercise training modulates glutamic acid decarboxylase-65/67 expression through TrkB signaling to ameliorate neuropathic pain in rats with spinal cord injury. Mol Pain. (2020) 16:1744806920924511. 10.1177/1744806920924511 32418502 PMC7235678

[86] Sliwinski C Nees TA Puttagunta R Weidner N Blesch A. Sensorimotor activity partially ameliorates pain and reduces nociceptive fiber density in the chronically injured spinal cord. J Neurotrauma. (2018) 35:2222–38. 10.1089/neu.2017.5431 29706124 PMC6119231

[87] Dugan EA Schachner B Jergova S Sagen J. Intensive locomotor training provides sustained alleviation of chronic spinal cord injury-associated neuropathic pain: a two-year pre-clinical study. J Neurotrauma. (2021) 38:789–802. 10.1089/neu.2020.7378 33218293

[88] Sanchez-Ventura J Gimenez-Llort L Penas C Udina E. Voluntary wheel running preserves lumbar perineuronal nets, enhances motor functions and prevents hyperreflexia after spinal cord injury. Exp Neurol. (2021) 336:113533. 10.1016/j.expneurol.2020.113533 33264633

[89] Endo T Ajiki T Inoue H Kikuchi M Yashiro T Nakama S. Early exercise in spinal cord injured rats induces allodynia through TrkB signaling. Biochem Biophys Res Commun. (2009) 381:339–44. 10.1016/j.bbrc.2009.02.043 19222991

[90] Detloff MR Quiros-Molina D Javia AS Daggubati L Nehlsen AD Naqvi A. Delayed exercise is ineffective at reversing aberrant nociceptive afferent plasticity or neuropathic pain after spinal cord injury in rats. Neurorehabil Neural Repair. (2016) 30:685–700. 10.1177/1545968315619698 26671215 PMC4907889

[91] Norrbrink C Lindberg T Wahman K Bjerkefors A. Effects of an exercise programme on musculoskeletal and neuropathic pain after spinal cord injury–results from a seated double-poling ergometer study. Spinal Cord. (2012) 50:457–61. 10.1038/sc.2011.160 22289901

[92] Ginis KM Latimer AE McKechnie K Ditor DS M'cCarney N Hicks AL. Using exercise to enhance subjective well-being among people with spinal cord injury: The mediating influences of stress and pain. Rehabil Psychol. (2003) 48:157–64. 10.1037/0090-5550.48.3.157

[93] Hicks AL Martin KA Ditor DS Latimer AE Craven C Bugaresti J. Long-term exercise training in persons with spinal cord injury: effects on strength, arm ergometry performance and psychological well-being. Spinal Cord. (2003) 41:34–43. 10.1038/sj.sc.3101389 12494319

[94] Ditor DS Latimer AE Ginis KA Arbour KP McCartney N Hicks AL. Maintenance of exercise participation in individuals with spinal cord injury: effects on quality of life, stress and pain. Spinal Cord. (2003) 41:446–50. 10.1038/sj.sc.3101487 12883542

[95] Fakhoury M. Spinal cord injury: overview of experimental approaches used to restore locomotor activity. Rev Neurosci. (2015) 26:397–405. 10.1515/revneuro-2015-0001 25870961

[96] Anwer S Equebal A Palekar TJ Nezamuddin M Neyaz O Alghadir A. Effect of locomotor training on motor recovery and walking ability in patients with incomplete spinal cord injury: a case series. J Phys Ther Sci. (2014) 26:951–3. 10.1589/jpts.26.951 25013303 PMC4085228

[97] Lo AC Triche EW. Improving gait in multiple sclerosis using robot-assisted, body weight supported treadmill training. Neurorehabil Neural Repair. (2008) 22:661–71. 10.1177/1545968308318473 18971381

[98] Knikou M. Plasticity of corticospinal neural control after locomotor training in human spinal cord injury. Neural Plast. (2012) 2012:254948. 10.1155/2012/254948 22701805 PMC3373155

[99] Hubscher CH Herrity AN Williams CS Montgomery LR Willhite AM Angeli CA. Improvements in bladder, bowel and sexual outcomes following task-specific locomotor training in human spinal cord injury. PLoS ONE. (2018) 13:e0190998. 10.1371/journal.pone.0190998 29385166 PMC5791974

[100] Hubscher CH Wyles J Gallahar A Johnson K Willhite A Harkema SJ. Effect of different forms of activity-based recovery training on bladder, bowel, and sexual function after spinal cord injury. Arch Phys Med Rehabil. (2021) 102:865–73. 10.1016/j.apmr.2020.11.002 33278365 PMC8084981

[101] Lofgren M Norrbrink C. “But I know what works”–patients' experience of spinal cord injury neuropathic pain management. Disabil Rehabil. (2012) 34:2139–47. 10.3109/09638288.2012.676146 22512334

[102] Todd KR Martin Ginis KA. An examination of diurnal variations in neuropathic pain and affect, on exercise and non-exercise days, in adults with spinal cord injury. Spinal Cord Ser Cases. (2018) 4:94. 10.1038/s41394-018-0130-3 30393565 PMC6204132

[103] Muraki S Tsunawake N Hiramatsu S Yamasaki M. The effect of frequency and mode of sports activity on the psychological status in tetraplegics and paraplegics. Spinal Cord. (2000) 38:309–14. 10.1038/sj.sc.3101002 10822404

[104] Lima LV Abner TSS Sluka KA. Does exercise increase or decrease pain? Central mechanisms underlying these two phenomena. J Physiol. (2017) 595:4141–50. 10.1113/JP273355 28369946 PMC5491894

[105] Harber VJ Sutton JR. Endorphins and exercise. Sports Med. (1984) 1:154–71. 10.2165/00007256-198401020-00004 6091217

[106] Basbaum AI Fields HL. Endogenous pain control systems: brainstem spinal pathways and endorphin circuitry. Annu Rev Neurosci. (1984) 7:309–38. 10.1146/annurev.ne.07.030184.001521 6143527

[107] Haier RJ Quaid K Mills JC. Naloxone alters pain perception after jogging. Psychiatry Res. (1981) 5:231–2. 10.1016/0165-1781(81)90052-4 6945616

[108] Olausson B Eriksson E Ellmarker L Rydenhag B Shyu BC Andersson SA. Effects of naloxone on dental pain threshold following muscle exercise and low frequency transcutaneous nerve stimulation: a comparative study in man. Acta Physiol Scand. (1986) 126:299–305. 10.1111/j.1748-1716.1986.tb07818.x 3486546

[109] Stagg NJ Mata HP Ibrahim MM Henriksen EJ Porreca F Vanderah TW. Regular exercise reverses sensory hypersensitivity in a rat neuropathic pain model: role of endogenous opioids. Anesthesiology. (2011) 114:940–8. 10.1097/ALN.0b013e318210f880 21386701 PMC6345518

[110] Brito RG Rasmussen LA Sluka KA. Regular physical activity prevents development of chronic muscle pain through modulation of supraspinal opioid and serotonergic mechanisms. Pain Rep. (2017) 2:e618. 10.1097/PR9.0000000000000618 29392233 PMC5777681

[111] Janal MN Colt EWD Clark CW Glusman M. Pain sensitivity, mood and plasma endocrine levels in man following long-distance running: effects of naloxone. Pain. (1984) 19:13–25. 10.1016/0304-3959(84)90061-7 6330643

[112] Droste C Greenlee MW Schreck M Roskamm H. Experimental pain thresholds and plasma beta-endorphin levels during exercise. Med Sci Sports Exerc. (1991) 23:334–42. 10.1249/00005768-199103000-00012 2020272

[113] Dey S Singh RH Dey PK. Exercise training: significance of regional alterations in serotonin metabolism of rat brain in relation to antidepressant effect of exercise. Physiol Behav. (1992) 52:1095–9. 10.1016/0031-9384(92)90465-E 1283013

[114] Brown BS Payne T Kim C Moore G Krebs P Martin W. Chronic response of rat brain norepinephrine and serotonin levels to endurance training. J Appl Physiol Respir Environ Exerc Physiol. (1979) 46:19–23. 10.1152/jappl.1979.46.1.19 457523

[115] Bobinski F Ferreira TAA Cordova MM Dombrowski PA da Cunha C Santo C. Role of brainstem serotonin in analgesia produced by low-intensity exercise on neuropathic pain after sciatic nerve injury in mice. Pain. (2015) 156:2595–2606. 10.1097/j.pain.0000000000000372 26447701 PMC4666012

[116] Koltyn KF Brellenthin AG Cook DB Sehgal N Hillard C. Mechanisms of exercise-induced hypoalgesia. J Pain. (2014) 15:1294–304. 10.1016/j.jpain.2014.09.006 25261342 PMC4302052

[117] Galdino G Romero T Pinho JF da Silva D Aguiar AM de Paula J Cruz. Acute resistance exercise induces antinociception by activation of the endocannabinoid system in rats. Anesth Analg. (2014) 119:702–15. 10.1213/ANE.0000000000000340 24977916 PMC4139418

[118] Galdino G Romero TR Silva JF Aguiar DC de Paula AM Cruz JS. The endocannabinoid system mediates aerobic exercise-induced antinociception in rats. Neuropharmacology. (2014) 77:313–24. 10.1016/j.neuropharm.2013.09.022 24148812

[119] Gleeson M Bishop NC Stensel DJ Lindley MR Mastana SS Nimmo MA. The anti-inflammatory effects of exercise: mechanisms and implications for the prevention and treatment of disease. Nat Rev Immunol. (2011) 11:607–15. 10.1038/nri3041 21818123

[120] Zhang JM An J. Cytokines, inflammation, and pain. Int Anesthesiol Clin. (2007) 45:27–37. 10.1097/AIA.0b013e318034194e 17426506 PMC2785020

[121] Davies AL Hayes KC Dekaban GA. Clinical correlates of elevated serum concentrations of cytokines and autoantibodies in patients with spinal cord injury. Arch Phys Med Rehabil. (2007) 88:1384–93. 10.1016/j.apmr.2007.08.004 17964877

[122] Hayes KC Hull TC Delaney GA Potter PJ Sequeira KA Campbell K. Elevated serum titers of proinflammatory cytokines and CNS autoantibodies in patients with chronic spinal cord injury. J Neurotrauma. (2002) 19:753–61. 10.1089/08977150260139129 12165135

[123] Arnold SA Hagg T. Anti-inflammatory treatments during the chronic phase of spinal cord injury improve locomotor function in adult mice. J Neurotrauma. (2011) 28:1995–2002. 10.1089/neu.2011.1888 21740131 PMC3172871

[124] Kigerl KA McGaughy VM Popovich PG. Comparative analysis of lesion development and intraspinal inflammation in four strains of mice following spinal contusion injury. J Comp Neurol. (2006) 494:578–94. 10.1002/cne.20827 16374800 PMC2655318

[125] Groah SL Nash MS Ljungberg IH Libin A Hamm LF Ward E. Nutrient intake and body habitus after spinal cord injury: an analysis by sex and level of injury. J Spinal Cord Med. (2009) 32:25–33. 10.1080/10790268.2009.11760749 19264046 PMC2647496

[126] Minihane AM Vinoy S Russell WR Baka A Roche HM Tuohy KM. Low-grade inflammation, diet composition and health: current research evidence and its translation. Br J Nutr. (2015) 114:999–1012. 10.1017/S0007114515002093 26228057 PMC4579563

[127] Ricker MA Haas WC. Anti-inflammatory diet in clinical practice: a review. Nutr Clin Pract. (2017) 32:318–25. 10.1177/0884533617700353 28350517

[128] Maiorino MI Bellastella G Petrizzo M Scappaticcio L Giugliano D Esposito K. Mediterranean diet cools down the inflammatory milieu in type 2 diabetes: the MEDITA randomized controlled trial. Endocrine. (2016) 54:634–41. 10.1007/s12020-016-0881-1 26860514

[129] Ramsden CE Faurot KR Zamora D Suchindran CM Macintosh BA Gaylord S. Targeted alteration of dietary n-3 and n-6 fatty acids for the treatment of chronic headaches: a randomized trial. Pain. (2013) 154:2441–51. 10.1016/j.pain.2013.07.028 23886520 PMC3850757

[130] Goldberg RJ Katz J. A meta-analysis of the analgesic effects of omega-3 polyunsaturated fatty acid supplementation for inflammatory joint pain. Pain. (2007) 129:210–23. 10.1016/j.pain.2007.01.020 17335973

[131] Kuptniratsaikul V Dajpratham P Taechaarpornkul W Buntragulpoontawee M Lukkanapichonchut P Chootip C. Efficacy and safety of Curcuma domestica extracts compared with ibuprofen in patients with knee osteoarthritis: a multicenter study. Clin Interv Aging. (2014) 9:451–8. 10.2147/CIA.S58535 24672232 PMC3964021

[132] Eller OC Foright RM Brake AD Winter MK Bantis LE Morris EM. An omega-3-rich anti-inflammatory diet improved widespread allodynia and worsened metabolic outcomes in adult mice exposed to neonatal maternal separation. Neuroscience. (2021) 468:53–67. 10.1016/j.neuroscience.2021.06.001 34107347 PMC8336378

[133] Bickford PC Gould T Briederick L Chadman K Pollock A Young D. Antioxidant-rich diets improve cerebellar physiology and motor learning in aged rats. Brain Res. (2000) 866:211–7. 10.1016/S0006-8993(00)02280-0 10825496

[134] Allison DJ Thomas A Beaudry K Ditor DS. Targeting inflammation as a treatment modality for neuropathic pain in spinal cord injury: a randomized clinical trial. J Neuroinflammation. (2016) 13:152. 10.1186/s12974-016-0625-4 27316678 PMC4912827

[135] Bailey KA Lenz K Allison DJ Ditor DS. Barriers and facilitators to adhering to an anti-inflammatory diet for individuals with spinal cord injuries. Health Psychol Open. (2018) 5:2055102918798732. 10.1177/2055102918798732 30202539 PMC6122254

[136] Freeman JM Kossoff EH Hartman AL. The ketogenic diet: one decade later. Pediatrics. (2007) 119:535–43. 10.1542/peds.2006-2447 17332207

[137] Kong G Liu J Li R Lin J Huang Z Yang Z. Ketone metabolite beta-hydroxybutyrate ameliorates inflammation after spinal cord injury by inhibiting the NLRP3 inflammasome. Neurochem Res. (2021) 46:213–29. 10.1007/s11064-020-03156-2 33108630

[138] Ruskin DN Sturdevant IC Wyss LS Masino SA. Ketogenic diet effects on inflammatory allodynia and ongoing pain in rodents. Sci Rep. (2021) 11:725. 10.1038/s41598-020-80727-x 33436956 PMC7804255

[139] Cooper MA Menta BW Perez-Sanchez C Jack MM Khan ZW Ryals JM. A ketogenic diet reduces metabolic syndrome-induced allodynia and promotes peripheral nerve growth in mice. Exp Neurol. (2018) 306:149–57. 10.1016/j.expneurol.2018.05.011 29763602 PMC5994385

[140] Masino SA Ruskin DN. Ketogenic diets and pain. J Child Neurol. (2013) 28:993–1001. 10.1177/0883073813487595 23680946 PMC4124736

[141] Yarar-Fisher C Kulkarni A Li J Farley P Renfro C Aslam H. Evaluation of a ketogenic diet for improvement of neurological recovery in individuals with acute spinal cord injury: a pilot, randomized safety and feasibility trial. Spinal Cord Ser Cases. (2018) 4:88. 10.1038/s41394-018-0121-4 30275980 PMC6155083

[142] Streijger F Plunet WT Lee JH Liu J Lam CK Park S. Ketogenic diet improves forelimb motor function after spinal cord injury in rodents. PLoS ONE. (2013) 8:e78765. 10.1371/journal.pone.0078765 24223849 PMC3817084

[143] Magge S Lembo A. Low-FODMAP diet for treatment of irritable bowel syndrome. Gastroenterol Hepatol. (2012) 8:739–45. PMC3966170 24672410

[144] Wong S Jamous A O'Driscoll J Sekhar R Weldon M Yau CY. A Lactobacillus casei Shirota probiotic drink reduces antibiotic-associated diarrhoea in patients with spinal cord injuries: a randomised controlled trial. Br J Nutr. (2014) 111:672–8. 10.1017/S0007114513002973 24044687

[145] Didari T Mozaffari S Nikfar S Abdollahi M. Effectiveness of probiotics in irritable bowel syndrome: Updated systematic review with meta-analysis. World J Gastroenterol. (2015) 21:3072–84. 10.3748/wjg.v21.i10.3072 25780308 PMC4356930

[146] Gareau MG Sherman PM Walker WA. Probiotics and the gut microbiota in intestinal health and disease. Nat Rev Gastroenterol Hepatol. (2010) 7:503–14. 10.1038/nrgastro.2010.117 20664519 PMC4748966

[147] Rondanelli M Faliva MA Miccono A Naso M Nichetti M Riva A. Food pyramid for subjects with chronic pain: foods and dietary constituents as anti-inflammatory and antioxidant agents. Nutr Res Rev. (2018) 31:131–51. 10.1017/S0954422417000270 29679994

[148] Holt EM Steffen LM Moran A Basu S Steinberger J Ross JA. Fruit and vegetable consumption and its relation to markers of inflammation and oxidative stress in adolescents. J Am Diet Assoc. (2009) 109:414–21. 10.1016/j.jada.2008.11.036 19248856 PMC2676354

[149] Miller ER Erlinger TP Sacks FM Svetkey LP Charleston J Li PH. A dietary pattern that lowers oxidative stress increases antibodies to oxidized LDL: results from a randomized controlled feeding study. Atherosclerosis. (2005) 183:175–82. 10.1016/j.atherosclerosis.2005.04.001 16216596

[150] Montonen J Boeing H Fritsche A Schleicher E Joost HG Schulze MB. Consumption of red meat and whole-grain bread in relation to biomarkers of obesity, inflammation, glucose metabolism and oxidative stress. Eur J Nutr. (2013) 52:337–45. 10.1007/s00394-012-0340-6 22426755 PMC3549403

[151] Daley CA Abbott A Doyle PS Nader GA Larson S. A review of fatty acid profiles and antioxidant content in grass-fed and grain-fed beef. Nutr J. (2010) 9:10. 10.1186/1475-2891-9-10 20219103 PMC2846864

[152] Maroon JC Bost JW. Omega-3 fatty acids (fish oil) as an anti-inflammatory: an alternative to nonsteroidal anti-inflammatory drugs for discogenic pain. Surg Neurol. (2006) 65:326–31. 10.1016/j.surneu.2005.10.023 16531187

[153] Gogebakan O Kohl A Osterhoff MA van Baak MA Jebb SA Papadaki A. Effects of weight loss and long-term weight maintenance with diets varying in protein and glycemic index on cardiovascular risk factors: the diet, obesity, and genes (DiOGenes) study: a randomized, controlled trial. Circulation. (2011) 124:2829–38. 10.1161/CIRCULATIONAHA.111.033274 22104550

[154] Veech RL. The therapeutic implications of ketone bodies: the effects of ketone bodies in pathological conditions: ketosis, ketogenic diet, redox states, insulin resistance, and mitochondrial metabolism. Prostaglandins Leukot Essent Fatty Acids. (2004) 70:309–19. 10.1016/j.plefa.2003.09.007 14769489

[155] Ruskin DN Kawamura M Masino SA. Adenosine and ketogenic treatments. J Caffeine Adenosine Res. (2020) 10:104–9. 10.1089/caff.2020.0011 32954218 PMC7499891

[156] Hasko G Cronstein B. Regulation of inflammation by adenosine. Front Immunol. (2013) 4:85. 10.3389/fimmu.2013.00085 23580000 PMC3619132

[157] Ravenscroft A Ahmed YS Burnside IG. Chronic pain after SCI. A patient survey. Spinal Cord. (2000) 38:611–4. 10.1038/sj.sc.3101073 11093322

[158] Tsai CY Bryce TN Delgado AD Mulroy S Maclntyre B Charlifue S. Treatments that are perceived to be helpful for non-neuropathic pain after traumatic spinal cord injury: a multicenter cross-sectional survey. Spinal Cord. (2021) 59:520–8. 10.1038/s41393-021-00621-9 33742116

[159] Khandia R Munjal AK Iqbal HMN Dhama K. Heat shock proteins: therapeutic perspectives in inflammatory disorders. Recent Pat Inflamm Allergy Drug Discov. (2017) 10:94–104. 10.2174/1872213X10666161213163301 27978789

[160] Granger DN Senchenkova E. Inflammation and the Microcirculation. San Rafael (CA): Morgan & Claypool Life Sciences (2010). 10.4199/C00013ED1V01Y201006ISP008 21452440

[161] Tracy B Armola R Micham J. The “cold cord”: a review of therapeutic hypothermia for traumatic spinal cord injuries. Am J Crit Care. (2015) 24:540–3. 10.4037/ajcc2015879 26523013

[162] Dietrich WD Levi AD Wang M Green BA. Hypothermic treatment for acute spinal cord injury. Neurotherapeutics. (2011) 8:229–39. 10.1007/s13311-011-0035-3 21416406 PMC3101829

[163] Yu CG Jimenez O Marcillo AE Weider B Bangerter K Dietrich WD. Beneficial effects of modest systemic hypothermia on locomotor function and histopathological damage following contusion-induced spinal cord injury in rats. J Neurosurg. (2000) 93:85–93. 10.3171/spi.2000.93.1.0085 10879763

[164] Vickers AJ Vertosick EA Lewith G MacPherson H Foster NE Sherman KJ. Acupuncture for chronic pain: update of an individual patient data meta-analysis. J Pain. (2018) 19:455–74. 10.1016/j.jpain.2017.11.005 29198932 PMC5927830

[165] Ernst E. Acupuncture–a critical analysis. J Intern Med. (2006) 259:125–37. 10.1111/j.1365-2796.2005.01584.x 16420542

[166] Nayak S Shiflett SC Schoenberger NE Agostinelli S Kirshblum S Averill A. Is acupuncture effective in treating chronic pain after spinal cord injury? Arch Phys Med Rehabil. (2001) 82:1578–86. 10.1053/apmr.2001.26624 11689979

[167] Guo J Zhu Y Yang Y Wang X Chen B Zhang W. Electroacupuncture at Zusanli (ST36) ameliorates colonic neuronal nitric oxide synthase upregulation in rats with neurogenic bowel dysfunction following spinal cord injury. Spinal Cord. (2016) 54:1139–44. 10.1038/sc.2016.76 27377302

[168] Wang J Zhai Y Wu J Zhao S Zhou J Liu Z. Acupuncture for chronic urinary retention due to spinal cord injury: a systematic review. Evid Based Complement Alternat Med. (2016) 2016:9245186. 10.1155/2016/9245186 27190542 PMC4846757

[169] Fan Q Cavus O Xiong L Xia Y. Spinal cord injury: how could acupuncture help? J Acupunct Meridian Stud. (2018) 11:124–32. 10.1016/j.jams.2018.05.002 29753705

[170] Da Silva MA Dorsher PT. Neuroanatomic and clinical correspondences: acupuncture and vagus nerve stimulation. J Altern Complement Med. (2014) 20:233–40. 10.1089/acm.2012.1022 24359451

[171] Borovikova LV Ivanova S Zhang M Yang H Botchkina GI Watkins LR. Vagus nerve stimulation attenuates the systemic inflammatory response to endotoxin. Nature. (2000) 405:458–62. 10.1038/35013070 10839541

[172] Tsaava T Datta-Chaudhuri T Addorisio ME Masi EB Silverman HA Newman JE. Specific vagus nerve stimulation parameters alter serum cytokine levels in the absence of inflammation. Bioelectron Med. (2020) 6:8. 10.1186/s42234-020-00042-8 32309522 PMC7146955

[173] Koopman FA Chavan SS Miljko S Grazio S Sokolovic S Schuurman PR. Vagus nerve stimulation inhibits cytokine production and attenuates disease severity in rheumatoid arthritis. Proc Natl Acad Sci USA. (2016) 113:8284–9. 10.1073/pnas.1605635113 27382171 PMC4961187

[174] Chen SL Wu XY Cao ZJ Fan J Wang M Owyang C. Subdiaphragmatic vagal afferent nerves modulate visceral pain. Am J Physiol Gastrointest Liver Physiol. (2008) 294:G1441–9. 10.1152/ajpgi.00588.2007 18420825 PMC3222235

[175] Chen H Feng Z Min L Deng W Tan M Hong J. Vagus nerve stimulation reduces neuroinflammation through microglia polarization regulation to improve functional recovery after spinal cord injury. Front Neurosci. (2022) 16:813472. 10.3389/fnins.2022.813472 35464311 PMC9022634

[176] Williams DA Cary MA Groner KH Chaplin W Glazer LJ Rodriguez AM. Improving physical functional status in patients with fibromyalgia: a brief cognitive behavioral intervention. J Rheumatol. (2002) 29:1280–6. 12064847

[177] Meissner K Schweizer-Arau A Limmer A Preibisch C Popovici RM Lange I. Psychotherapy with somatosensory stimulation for endometriosis-associated pain: a randomized controlled trial. Obstet Gynecol. (2016) 128:1134–42. 10.1097/AOG.0000000000001691 27741200

[178] Eccleston C Fisher E Craig L Duggan GB Rosser BA Keogh E. Psychological therapies (Internet-delivered) for the management of chronic pain in adults. Cochrane Database Syst Rev. (2014) 2014:CD010152. 10.1002/14651858.CD010152.pub2 24574082 PMC6685592

[179] Hilton L Hempel S Ewing BA Apaydin E Xenakis L Newberry S. Mindfulness meditation for chronic pain: systematic review and meta-analysis. Ann Behav Med. (2017) 51:199–213. 10.1007/s12160-016-9844-2 27658913 PMC5368208

[180] Volodina M Smetanin N Lebedev M Ossadtchi A. Cortical and autonomic responses during staged Taoist meditation: two distinct meditation strategies. PLoS ONE. (2021) 16:e0260626. 10.1371/journal.pone.0260626 34855823 PMC8638869

[181] Paul SP Basude D. Non-pharmacological management of abdominal pain-related functional gastrointestinal disorders in children. World J Pediatr. (2016) 12:389–98. 10.1007/s12519-016-0044-8 27363985

[182] Flor H Fydrich T Turk DC. Efficacy of multidisciplinary pain treatment centers: a meta-analytic review. Pain. (1992) 49:221–30. 10.1016/0304-3959(92)90145-2 1535122

[183] Waters SJ McKee DC Keefe FJ. Cognitive behavioral approaches to the treatment of pain. Psychopharmacol Bull. (2007) 40:74–88. 18227779

[184] Kennedy P Duff J Evans M Beedie A. Coping effectiveness training reduces depression and anxiety following traumatic spinal cord injuries. Br J Clin Psychol. (2003) 42:41–52. 10.1348/014466503762842002 12675978

[185] Craig AR Hancock K Chang E Dickson H. Immunizing against depression and anxiety after spinal cord injury. Arch Phys Med Rehabil. (1998) 79:375–7. 10.1016/S0003-9993(98)90136-8 9552101

[186] Norrbrink Budh C Kowalski J Lundeberg T. A comprehensive pain management programme comprising educational, cognitive and behavioural interventions for neuropathic pain following spinal cord injury. J Rehabil Med. (2006) 38:172–80. 10.1080/16501970500476258 16702084

[187] Perry KN Nicholas MK Middleton JW. Comparison of a pain management program with usual care in a pain management center for people with spinal cord injury-related chronic pain. Clin J Pain. (2010) 26:206–16. 10.1097/AJP.0b013e3181bff8f3 20173434

[188] Heutink M Post MWM Bongers-Janssen HMH Dijkstra CA Snoek GJ Spijkerman DCM. The CONECSI trial: results of a randomized controlled trial of a multidisciplinary cognitive behavioral program for coping with chronic neuropathic pain after spinal cord injury. Pain. (2012) 153:120–8. 10.1016/j.pain.2011.09.029 22100355

[189] Burns AS Delparte JJ Ballantyne EC Boschen KA. Evaluation of an interdisciplinary program for chronic pain after spinal cord injury. PM R. (2013) 5:832–8. 10.1016/j.pmrj.2013.05.004 23684779

[190] Hearn JH Cross A. Mindfulness for pain, depression, anxiety, and quality of life in people with spinal cord injury: a systematic review. BMC Neurol. (2020) 20:32. 10.1186/s12883-020-1619-5 31964353 PMC6971852

[191] Zanca JM Gilchrist C Ortiz CE Dyson-Hudson TA. Pilot clinical trial of a clinical meditation and imagery intervention for chronic pain after spinal cord injury. J Spinal Cord Med. (2021) 1–15. 10.1080/10790268.2021.1970894 34612802 PMC9135436

[192] Chalageri E Vishwakarma G Ranjan RL Govindaraj R Chhabra HS. Effect of Raja Yoga meditation on psychological and functional outcomes in spinal cord injury patients. Int J Yoga. (2021) 14:36–42. 10.4103/ijoy.IJOY_68_20 33840975 PMC8023440

[193] Lilley E Andrews MR Bradbury EJ Elliott H Hawkins P Ichiyama RM. Refining rodent models of spinal cord injury. Exp Neurol. (2020) 328:113273. 10.1016/j.expneurol.2020.113273 32142803

[194] Sorge RE Totsch SK. Sex differences in pain. J Neurosci Res. (2017) 95:1271–81. 10.1002/jnr.23841 27452349

